# Comparison of Trends and Complications of Unicompartmental Knee Arthroplasty Versus Periarticular Knee Osteotomy Among ABOS Part II Oral Examination Candidates

**DOI:** 10.1177/23259671241257818

**Published:** 2024-07-31

**Authors:** Kylie T. Callan, Eric Smith, Theofilos Karasavvidis, Dean Wang

**Affiliations:** * Department of Orthopaedic Surgery, University of California–Irvine Health, Orange, California, USA; Investigation performed at University of California–Irvine Health, Orange, California, USA

**Keywords:** cartilage, knee arthroplasty, osteotomy, fellowship

## Abstract

**Background:**

While unicompartmental knee arthroplasty (UKA) and osteotomy procedures are commonly used to treat knee osteoarthritis, the differences in complication profiles between procedures are still poorly understood.

**Purpose/Hypothesis:**

The purpose of this study was to assess the trends and complication rates of UKA and periarticular knee osteotomy for knee osteoarthritis among newly trained surgeons by using the American Board of Orthopaedic Surgery (ABOS) Part II Oral Examination Case List database. It was hypothesized that more adult reconstruction fellowship-trained surgeons would perform UKA, while more sports medicine fellowship-trained surgeons would perform osteotomy, and that both procedures would have low rates of complications.

**Study Design:**

Cross-sectional study; Level of evidence, 3.

**Methods:**

The ABOS database was queried for patients who underwent UKA, high tibial osteotomy, and distal femoral osteotomy procedures in examination years 2011 to 2021. Patient characteristics, surgeon fellowship training history, surgeon-reported postoperative complications, and readmission and reoperation rates were recorded. Comparisons between the UKA and osteotomy groups were performed using independent *t* tests and chi-square tests.

**Results:**

There were 2524 patients in the UKA group and 270 patients in the osteotomy group. The majority of newly trained surgeons performing UKA (70.5%) had fellowship training in adult reconstruction, while the majority of those performing osteotomy (57.8%) had fellowship training in sports medicine (*P* < .001). The incidence of UKA and osteotomy increased during the study period (18.8 UKAs and 1.8 osteotomies performed per 10,000 cases in 2011 vs 39.5 UKAs and 4.2 osteotomies performed per 10,000 cases in 2021). Rates were significantly higher for osteotomy compared with UKA regarding anesthetic complications (2.2% vs 0.6%; *P* = .015), surgical complications (23.7% vs 7.3%; *P* < .001), reoperation (5.2% vs 1.9%; *P* = .002), and infection (6.7% vs 1.4%; *P* < .001). There were no significant differences in rates of medical complication, readmission, deep vein thrombosis, pulmonary embolism, or stiffness/arthrofibrosis.

**Conclusion:**

Among newly trained surgeons taking the ABOS Part II Oral Examination, the incidence of UKA and periarticular knee osteotomy increased over the past decade. Compared with UKA, complication rates were higher after osteotomy, with an overall surgical complication rate of 23.7%.

Knee osteoarthritis (OA) is a prevalent cause of knee pain and dysfunction.^1^ While it can affect all 3 knee compartments, up to one-third of patients with knee OA have been found to have primarily unicompartmental OA.^4,12,27^ Unicompartmental knee arthroplasty (UKA), high tibial osteotomy (HTO), and distal femoral osteotomy (DFO) are 3 well-established treatment modalities intended to treat unicompartmental OA. By preservation of the nonaffected compartments, ligaments, and knee kinematics, UKA has been associated with shorter operative time, shorter hospital length of stay, greater postoperative range of motion, shorter recovery, and higher Forgotten Joint Score when compared with total knee arthroplasty (TKA).^7,14,25^ As opposed to resurfacing damaged articular cartilage, osteotomy treatments such as HTO and DFO aim to correct knee malalignment, thereby offloading the arthritic compartment.^10,14,17^ HTO was first introduced in the 1970s to correct limb malalignment, unload the affected compartment, and preserve native cartilage in patients with OA with deformities of the tibia.^13^ Additionally, DFO is typically used to correct genu valgum deformity and/or patellofemoral maltracking and unload contact pressures in patients with lateral compartment OA.^20^

Although all 3 procedures are commonly performed to treat select patients with unicompartmental OA, the modern complication rates and profiles of these procedures are still poorly understood, particularly with recent advances in their surgical methods, implant materials, and rehabilitation. Historically, some groups have reported that UKA has lower revision rates compared with osteotomies, while others have shown no differences in rates of conversion to TKA.^4,6,19^ In some studies, UKA has been associated with fewer complications, better functional outcomes, and less postoperative pain, while HTO has been associated with greater range of motion.^4,6,19^ One study comparing UKA and DFO showed similar improvement in knee scores after surgery.^17^ Other studies exclusively examining DFO procedures have shown increased healing times and reoperation rates by over 50%.^3,8^ Jeon et al showed no significant differences in rates of postoperative complication between HTO and DFO in a study examining 47 patients with knee OA.^9^ Additionally, Yim et al^28^ compared the 2 procedures and saw similar functional outcomes and a rate of complications of about 6% in both groups. A study by our author group^11^ using the American College of Surgeons National Surgical Quality Improvement Project (ACS-NSQIP) database to review patients who underwent HTO and UKA between 2006 and 2019 found that there were no statistically significant differences in 30-day complication rates of venous thromboembolism, surgical site infection, and reoperation.

Currently, any differences in complication profiles between modern osteotomy and UKA procedures have not been investigated in detail. A better understanding of the complication profiles could assist physicians in clinical decision-making and patient counseling. In addition, although both the rates of UKA and the number of surgeons performing UKA have increased over the past decade, there are few studies exploring the association between a surgeon's training background and outcomes after UKA, HTO, and DFO.^5^

In this study, the American Board of Orthopaedic Surgery (ABOS) Part II Oral Examination Case List database was used to evaluate the trends and complication rates after UKA and osteotomy (HTO and DFO) procedures for unicompartmental knee OA among newly trained surgeons taking the ABOS Part II Oral Examination. The hypotheses were that (1) UKA would be performed by a higher percentage of adult reconstruction fellowship-trained surgeons, while osteotomy would be performed by a higher percentage of sports medicine fellowship-trained surgeons, and (2) both UKA and osteotomy would have low rates of complications and revision surgery.

## Methods

### Participants

This was a cross-sectional study of newly trained surgeons who performed UKA, HTO, and DFO and reported the procedures for the ABOS Part II Oral Examination. This study was deemed exempt from institutional review board approval due to the de-identification of patient and surgeon information. A research proposal and request for data were submitted to the ABOS Research Committee, and approval was granted.

The ABOS database includes all case lists submitted for review by ABOS Part II candidates, which consist of all surgical cases performed over a 6-month collection period in preparation for the oral examination portion of their ABOS board certification process. This database has been compiled annually from 1999 onward, with the unexpected reoperation and readmission variables available between 2013 and the present.

The ABOS database was queried between 2011 and 2021 for all candidates who logged at least 1 of the following procedures as identified by their Current Procedural Terminology (CPT) codes: UKA (CPT 27446), DFO (CPT 27450), and HTO (CPT 27457). Pediatric patients (aged <15 years) were excluded. Diagnoses were examined for all patients who underwent an osteotomy using International Classification of Diseases (ICD) codes. Patients were excluded if they had diagnoses unrelated to knee OA or cartilage degeneration (eg, isolated fractures, contractures, metabolic disorders, complications of prosthetic devices, or congenital deformities), without concurrent cartilage degeneration diagnoses.

**Figure 1.**
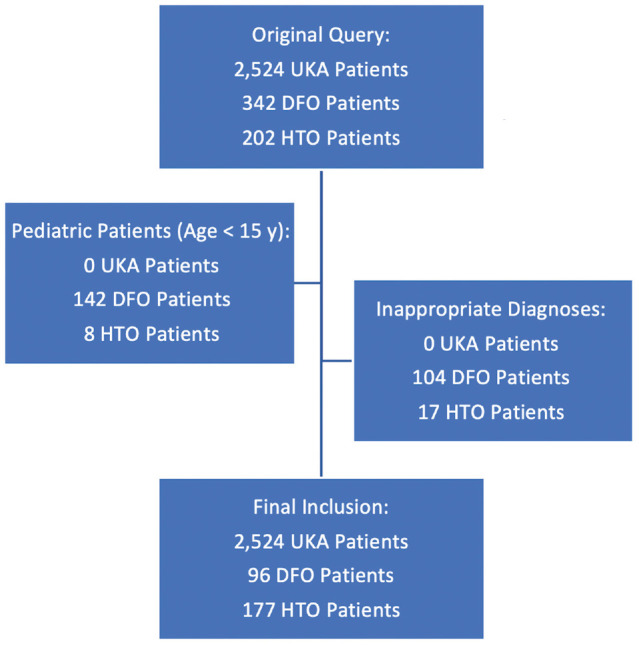
Flowchart illustrating inclusion and exclusion criteria for patients in the database. DFO, distal femoral osteotomy; HTO, high tibial osteotomy; UKA, unicompartmental knee arthroplasty.

The original query returned 2524 patients who underwent UKA, 342 patients who underwent DFO, and 202 patients who underwent HTO. Of these, 2524 patients treated with UKA and 270 patients treated with an osteotomy (93 with DFO, 174 with HTO, and 3 patients with both) were included in the final analysis (Figure 1).

### Data Collection

Patient characteristics, intraoperative data, and surgeon fellowship training history were collected. Surgeon-reported postoperative 90-day complications, including general anesthetic, medical, and surgical complications; rates of readmission; and rates of reoperation, were also recorded. Anesthetic complications included block anesthetic complications and general anesthetic complications. Medical complications included the following: anemia, arrhythmia, cerebral vascular accident, confusion/delirium, congestive heart failure, deep venous thrombosis (DVT), dermatologic complaint, gastrointestinal bleeding/ulcer/gastritis, hypotension, hypoxia/shortness of breath, medical unspecified, medication error/reaction, myocardial infarction, pneumonia, pulmonary embolism (PE), renal failure, respiratory failure, urinary retention, and urinary tract infection. Surgical complications included bone fracture, dislocation, fall, hemarthrosis/effusion, hematoma/seroma, implant failure/fracture/malfunction, infection, loss of reduction, nerve palsy/injury, nonunion/delayed union, pain, reflex sympathetic dystrophy/complex regional pain syndrome, skin ulcer/blister, stiffness/arthrofibrosis, surgical procedure intervention, surgical unspecified, tendon/ligament injury, vascular injury, wound dehiscence, and wound-healing delay/failure.

The primary outcomes assessed were rates of reoperation, readmission, DVT, PE, infection, and stiffness/arthrofibrosis after surgery. Secondary outcomes included overall anesthetic complications, overall medical complications, and overall surgical complications.

### Statistical Analysis

All categorical variables were reported as absolute values and percentages, and continuous variables were reported as means and standard deviations. Comparisons between UKA and osteotomy groups were performed using independent *t* tests and chi-square tests, as appropriate, with GraphPad Prism 9.4 (GraphPad Software Inc). These same analyses were performed within the osteotomy group to compare the DFOs and HTOs. Significance was set at *P* < .05.

## Results

### Patient Characteristics

Male patients comprised 47.0% of the UKA group (1186 of 2524 patients) and 56.3% of the osteotomy group 152 of 270 patients (*P* = .004). Patients who underwent osteotomy were significantly younger than those who underwent UKA (mean, 32.6 ± 12.1 years [interquartile range, 15-69 years] vs 62.1 ± 10.5 years [interquartile range, 23-91 years]; *P* < .001). Concurrent procedures were performed in 76.3% of patients in the osteotomy group, while concurrent procedures were performed in 7.8% of patients in the UKA group (*P* < .001). In the osteotomy group, these included autologous chondrocyte implantation (n = 8), osteochondral allografts (n = 25) and autografts (n = 2), ligamentous reconstructions (n = 10), meniscal transplants (n = 4), removal of loose or foreign bodies (n = 8), debridement of cartilage (n = 29), microfracture (n = 9), meniscal repair procedures (n = 8), and removal of osteochondritis dissecans lesions (n = 2).

### Trends in Fellowship Subspecialty Training and Annual Case Numbers

The majority of newly trained surgeons performing UKA had fellowship training in adult reconstruction (70.5%), while most newly trained surgeons performing osteotomy had fellowship training in sports medicine (57.8%) (*P* < .001). The incidence of UKA and osteotomy gradually increased during the study period. In examination year 2011, 18.8 UKAs and 1.8 osteotomies were performed per 10,000 cases, whereas in 2021, 39.5 UKAs and 4.2 osteotomies were performed per 10,000 cases (Table 1).

**Table 1:** Trends in UKA and Osteotomy Incidence, ABOS Part II Oral Examination Years 2011-2021^*a*^

Year	UKA (n = 2524)	Osteotomy (n = 270)	Total Cases, n
2011	145 (0.188)	14 (0.018)	77,158
2012	202 (0.227)	21 (0.024)	88,889
2013	137 (0.163)	35 (0.042)	83,817
2014	128 (0.141)	26 (0.029)	90,525
2015	275 (0.305)	20 (0.022)	90,045
2016	177 (0.214)	16 (0.019)	82,639
2017	188 (0.217)	18 (0.021)	86,648
2018	201 (0.232)	15 (0.017)	86,712
2019	346 (0.390)	28 (0.032)	88,763
2020	318 (0.363)	34 (0.039)	87,693
2021	407 (0.395)	43 (0.042)	102,914

a Data are reported as n (%) of total cases in that year unless otherwise indicated. ABOS, American Board of Orthopaedic Surgery; UKA, unicompartmental knee arthroplasty.

### Complication Rates

Surgical complications for UKA, osteotomy, DFO (n = 96), and HTO (n = 177) are shown in Table 2. The most common complications in the UKA group were wound-healing delay/failure (1.47%), infection (1.35%), pain (0.951%), hematoma/seroma (0.475%), and stiffness/arthrofibrosis (0.475%). The most common complications in the osteotomy group were infection (6.67%), bone fracture (3.70%), nonunion/delayed union (2.59%), nerve palsy/injury (2.22%), and implant failure/fracture/malfunction (1.85%) (Table 2). No significant differences in rates of medical complication, readmission, DVT, PE, or stiffness/arthrofibrosis were identified between the UKA and osteotomy groups (Table 3). When compared with the UKA group, the osteotomy group was associated with higher rates of anesthetic complications (2.2% vs 0.6%; *P* = .015), surgical complications (23.7% vs 7.3%; *P* < .001), reoperation (5.2% vs 1.9%; *P* = .002), and infection (6.7% vs 1.4%; *P* < .001) (Table 3). When examining surgical complications in cases with no concomitant procedures, there remained a significantly higher rate of surgical complications in isolated osteotomies as compared with isolated UKAs (26.6% of isolated osteotomies vs 6.8% of isolated UKAs; *P* < .001). There were no statistically significant differences in complication rates when comparing isolated osteotomies with osteotomies with concomitant procedures (26.6% vs 22.8%, respectively; *P* = .614).

**Table 2:** Surgical Complications for UKA, DFO, HTO, and Osteotomy^*a*^

Complication	UKA (n = 2524)	DFO (n = 96)^*b*^	HTO (n = 177)^*b*^	Osteotomy (n = 270)
Bone fracture	10 (0.396)	5 (5.21)	6 (3.39)	10 (3.70)
Dislocation	0 (0)	1 (1.04)	0 (0)	1 (0.370)
Fall	9 (0.357)	3 (3.13)	1 (0.565)	4 (1.48)
Hemarthrosis/effusion	10 (0.396)	0 (0)	1 (0.565)	1 (0.370)
Hematoma/seroma	12 (0.475)	0 (0)	3 (1.70)	3 (1.11)
Implant failure/fracture/malfunction	8 (0.317)	2 (2.08)	3 (1.70)	5 (1.85)
Infection	34 (1.35)	3 (3.13)	15 (8.48)	18 (6.67)
Loss of reduction	0 (0)	0 (0)	2 (1.13)	2 (0.741)
Nerve palsy/injury	4 (0.158)	2 (2.08)	4 (2.26)	6 (2.22)
Nonunion/delayed union	0 (0)	4 (4.17)	3 (1.70)	7 (2.59)
Pain, recurrent/persistent/uncontrolled	24 (0.951)	0 (0)	1 (0.565)	1 (0.370)
Skin ulcer/blister	11 (0.436)	1 (1.04)	3 (1.70)	4 (1.48)
Stiffness/arthrofibrosis	12 (0.475)	1 (1.04)	2 (1.13)	3 (1.11)
Surgical procedure intervention	1 (0.040)	0 (0)	1 (0.565)	1 (0.370)
Surgical unspecified	30 (1.19)	1 (1.04)	5 (2.83)	6 (2.22)
Tendon/ligament injury	5 (0.198)	0 (0)	0 (0)	0 (0)
Vascular injury	0 (0)	0 (0)	1 (0.565)	1 (0.370)
Wound dehiscence	2 (0.079)	0 (0)	1 (0.565)	1 (0.370)
Wound-healing delay/failure	37 (1.47)	0 (0)	4 (2.26)	4 (1.48)
Reflex sympathetic dystrophy/complex regional pain syndrome	2 (0.079)	0 (0)	0 (0)	0 (0)

a Data are reported as n (%). DFO, distal femoral osteotomy; HTO, high tibial osteotomy; UKA, unicompartmental knee arthroplasty.

b Three patients had both DFO and HTO.

**Table 3:** UKA Versus Osteotomy: Complications and Characteristics^*a*^

Variable	UKA (n = 2524)	Osteotomy (n = 270)	*P*
Complication rate, %			
Anesthetic complications	0.634	2.22	**.015**
Medical complications	4.24	3.70	.873
Surgical complications	7.25	23.7	**<.001**
Reoperation	1.90	5.19	**.002**
Readmission	1.98	2.96	.262
Infection	1.35	6.67	**<.001**
DVT	0.277	1.11	.064
PE	0.317	1.11	.082
Stiffness/arthrofibrosis	0.475	1.11	.171
Surgeon training, %			**<.001**
Adult reconstruction	70.5%	13.7	
Sports medicine	15.9%	57.8	
Other	15.1%	30.7	
Male sex, %	47.0%	56.3	**.004**
Age, y			**<.001**
Mean ± SD	62.1 ± 10.5	32.6 ± 12.1	
Range	55.0-70.0	21.8-42.0	
Interquartile range	23.0-91.0	15.0-69.0	

a Boldface *P* values indicate a statistically significant difference between groups (*P* < .05). DVT, deep venous thrombosis; PE, pulmonary embolism; UKA, unicompartmental knee arthroplasty.

### Comparison of HTO Versus DFO

Male patients comprised 38.5% of the DFO group and 66.1% of the HTO group (*P* < .001). The mean patient age was 25.7 ± 11.7 years (interquartile range, 15-68 years) in the DFO group and 36.1 ± 10.8 years (interquartile range, 15-69 years) in the HTO group (*P* < .001). According to training background, 44.8% of newly trained surgeons performing DFO had completed a sports medicine fellowship, while 63.8% of those performing HTO had sports medicine fellowship training (*P* = .006). There were no significant differences in any complication rates between the DFO and HTO procedures (Table 4). The most common surgical complications in the DFO group were bone fracture (5.2%), nonunion/delayed union (4.2%), infection (3.1%), and fall (3.1%). In comparison, the most common surgical complications in the HTO group were infection (8.5%), bone fracture (3.4%), nerve palsy/injury (2.3%), and wound-healing delay/failure (2.3%) (Table 2).

**Table 4:** DFO Versus HTO: Complications and Characteristics^*a*^

Variable	DFO (n = 96)	HTO (n = 177)	*P*
Complication rate, %			
Anesthetic complications	2.08	2.26	>.999
Medical complications	1.04	5.08	.173
Surgical complications	19.8	26.0	.298
Reoperation	5.21	5.08	>.999
Readmission	3.13	2.82	>.999
Infection	3.13	8.47	.125
DVT	0	1.69	.554
PE	0	1.69	.554
Stiffness/arthrofibrosis	1.04	1.13	>.999
Surgeon training, %			**.006**
Adult reconstruction	15.6	12.4	
Sports medicine	44.8	63.8	
Neither	42.7	25.4	
Male sex, %	38.5	66.1	**<.001**
Age, y			**<.001**
Mean ± SD	25.7 ± 11.7	36.1 ± 10.8	
Range	16.0-34.0	29.0-43.0	
Interquartile range	15.0-68.0	15.0-69.0	

a Three patients had both DFO and HTO. Boldface *P* values indicate a statistically significant difference between groups (*P* < .05). DFO, distal femoral osteotomy; DVT, deep venous thrombosis; HTO, high tibial osteotomy; PE, pulmonary embolism.

## Discussion

In this analysis of ABOS Part II Oral Examination candidates between 2011 and 2021, most newly trained surgeons performing UKA for knee OA had completed a fellowship in adult reconstruction (70.5%), and most newly trained surgeons performing osteotomy for knee OA had fellowship training in sports medicine (57.8%) (*P* < .001). The incidence of UKA and osteotomy gradually increased between 2011 (18.8 UKAs and 1.8 osteotomies performed per 10,000 cases) and 2021 (39.5 UKAs and 4.2 osteotomies performed per 10,000 cases). Rates of anesthetic complications, surgical complications, reoperation, and infection were significantly higher for osteotomy compared with UKA (anesthetic: 2.2% vs 0.6%, *P* = .015; surgical: 23.7% vs 7.3%, *P* < .001; reoperation: 5.2% vs 1.9%, *P* = .002; infection: 6.7% vs 1.4%; *P* < .001). Contrary to our hypothesis, the overall self-reported surgical complication rate after osteotomy was relatively high (23.7%). There were no significant differences in rates of medical complication, readmission, DVT, PE, or stiffness/arthrofibrosis between the UKA and osteotomy groups. Within the osteotomy group, there were no significant differences between DFO and HTO procedures in rates of anesthetic complications, medical complications, surgical complications, reoperation, readmission, infection, DVT, PE, or stiffness/arthrofibrosis.

There are limited data investigating the degree to which specific fellowship training influences the rate of performance of UKA versus osteotomy procedures. However, the results in this study of most surgeons performing UKA having fellowship training in adult reconstruction and most surgeons performing osteotomy having fellowship training in sports medicine support our study hypothesis. It is understandable that a larger proportion of those performing arthroplasty procedures, including UKA, would be fellowship-trained in adult reconstruction. Likewise, osteotomy procedures are well-established joint-preserving treatment modalities of deformity, soft tissue pathology, and arthritis and are part of the orthopaedic sports medicine armamentarium.^15^

The increasing incidence of both UKA and osteotomy in the study period aligns with previous findings. Carender and colleagues^5^ also used the ABOS Part II Oral Examination Case List database to examine the trends in UKA usage between 2010 and 2019 and found that rates of UKA increased, but rates of UKA compared with TKA remained about the same over the study period. They attributed these trends to an increasing proportion of surgeons pursuing adult reconstruction fellowships.^5^ This may also be due to improving technology, increased surgeon comfort with the procedure, and increased shared decision-making between the physician and patient. Based on data between 2000 and 2014, the rate of TKA in the United States is expected to increase by 85% by 2030.^21^ A similar study from New Zealand projected that the number of TKA procedures performed will nearly double between 2013 and 2038.^26^ Coupled with the findings of Carender et al^5^ that UKA and TKA trend together, it is reasonable to expect similar increases in the incidence of UKA and osteotomy in the future.

Regarding complication rates, Cao et al^4^ in their review and meta-analysis of 5 studies^9,16,22-24^ evaluated 394 patients with medial compartment OA who underwent either UKA or HTO and found significantly fewer complications in the UKA group compared with the HTO group, which is consistent with the results of the current study. In terms of overall rate of complications, we saw a surgical complication rate of 23.7% in the osteotomy group (19.8% in the DFO group and 26.0% in the HTO group) and 7.3% in the UKA group. Cao et al^4^ saw a rate of 11.7 complications per 100 cases in their HTO group (range for individual studies, 4.3-28.1) and 5.05 surgical complications per 100 cases in their UKA group (range for individual studies, 0.0-10.0). Therefore, the results of this study fall within the range of the studies^9,16,22-24^ included in the Cao et al^4^ review. It is worth noting that the study with a rate of 28.1 complications per 100 cases was the only randomized controlled trial of the 5 studies.^22^ Some of the differences in the complication rates may be attributed to the fact that all the studies mentioned different sets of surgical complications.

The complication rates in this study for both UKA and osteotomy were higher than would be expected for more experienced surgeons, illustrating the learning curves for these procedures after the completion of fellowship training. A recent multicenter study analyzing surgeons in their first post-fellowship year suggested that surgical complications of anterior total hip replacements normalize after about 40 cases.^2^ It would be reasonable to hypothesize that a similar or steeper curve exists for UKA and periarticular knee osteotomy. In particular, newly trained surgeons likely had increased operative times for osteotomy, which may be correlated with the increased rate of infections in HTOs, where the proximal medial tibial soft tissue envelope is thin.

Cao et al^4^ also examined reoperation rates and found a lower revision rate in the UKA group than in the HTO group, which is consistent with the findings of the current study. A retrospective cohort study examining 270 patients who underwent UKA and 113 patients who underwent HTO from the Military Health System found a significantly higher rate of conversion to TKA in patients who underwent UKA.^18^ Revision arthroplasties occurred at a mean of 2.9 years after surgery for the HTO group and 3.1 years after surgery for the UKA group, which is outside the time frame of follow-up for patients in the current study's database. Our previous study^11^ comparing complications of UKA versus HTO using the ACS-NSQIP database showed that HTO was associated with a longer operating time and higher rate of superficial infection but saw no significant differences in thromboembolism, urinary tract infection, transfusion, deep infection, or reoperation. This is consistent with our findings of no significant differences in rates of DVT and PE but counter to our findings of an increased reoperation rate in the osteotomy group.

### Limitations

The study results should be interpreted in the context of the following limitations. First, this was a retrospective, large database study involving demographically different patient groups. Second, as with any database study, despite standardization practices, it may have been unduly influenced by errors in coding or underreporting of complications. Additionally, this study could only assess the complications that were self-reported by earlycareer orthopaedic surgeons in the database. These may have been limited by the short follow-up time (typically <1 year), and complications may have fallen outside the predetermined categories for reporting. This short follow-up limited comparisons of more long-term complications such as conversion to TKA, which generally occurs at later postoperative time points. The degree of severity of each complication was also not reported in the database. Similarly, each case is indexed only by CPT and ICD codes, so inclusion and exclusion criteria could not be more nuanced. This limitation may be especially relevant for younger patients, for whom cartilage defects may be more multifactorial than in older, stereotypical patients with OA. Additionally, we could not effectively determine whether procedures were used as staging operations, especially for osteotomy procedures. This also means it was not possible to distinguish between lateral and medial UKA procedures. As lateral UKA procedures are more technically difficult, these may have increased complication rates as compared with medial UKA procedures. Last, while pooling data from such a large number of surgeons at various institutions increases the number of patients who can be assessed, there is heterogeneity in the practice environment, sterilization technique, and operative indications, which may have affected the complication rates.

## Conclusion

Among newly trained surgeons taking the ABOS Part II Oral Examination, the incidence of UKA and periarticular knee osteotomies has increased over the last decade. Compared with UKA, complication rates were higher after osteotomy, with an overall surgical complication rate of 23.7%. There were no significant differences in rates of medical complication, readmission, DVT, PE, or stiffness/arthrofibrosis between UKA and periarticular knee osteotomy.
